# The Impact of a Non-Pathogenic Strain of Fusarium Oxysporum on Structural and Biochemical Properties of Flax Suspension Cultures

**DOI:** 10.3390/ijms25179616

**Published:** 2024-09-05

**Authors:** Magdalena Wróbel-Kwiatkowska, Aleksandra Osika, Justyna Liszka, Mateusz Lipiński, Lucyna Dymińska, Michał Piegza, Waldemar Rymowicz

**Affiliations:** 1 Department of Biotechnology and Food Microbiology, Faculty of Biotechnology and Food Science, Wrocław University of Environmental and Life Sciences, 51-630 Wrocław, Polandmichal.piegza@upwr.edu.pl (M.P.); waldemar.rymowicz@upwr.edu.pl (W.R.); 2 Department of Bioorganic Chemistry, Wroclaw University of Economics and Business, 53-345 Wrocław, Poland; lucyna.dyminska@ue.wroc.pl

**Keywords:** *Linum usitatissimum* L., Fo47, FTIR, flavonoids, phenolic compounds

## Abstract

Flax (*Linum usitatissimum* L.) is an important crop plant with pharmaceutical significance. It is described in pharmacopoeias (the United States Pharmacopeia and the European Pharmacopoeia), which confirms that it (especially the seeds) is a valuable medicinal product. Similar to flax seeds, which accumulate bioactive compounds, flax in vitro cultures are also a rich source of flavonoids, phenolics, lignans and neolignans. In the present study, flax suspension cultures after treatment of the non-pathogenic *Fusarium oxysporum* strain Fo47 were established and analyzed. The study examined the suitability of Fo47 as an elicitor in flax suspension cultures and provided interesting data on the impact of these endophytic fungi on plant metabolism and physiology. Two flax cultivars (Bukoz and Nike) and two compositions of media for flax callus liquid cultures were tested. Biochemical analysis revealed enhanced levels of secondary metabolites (total flavonoid and total phenolic content) and photosynthetically active pigments in the flax callus cultures after treatment with the non-pathogenic fungal strain *F. oxysporum* Fo47 when compared to control, untreated cultures. In cultures with the selected, optimized conditions, FTIR analysis was performed and revealed changes in the structural properties of cell wall polymers after elicitation of cultures with *F. oxysporum* Fo47. The plant cell wall polymers were more strongly bound, and the crystallinity index (Icr) of cellulose was higher than in control, untreated samples. However, lignin and pectin levels were lower in the flax callus liquid cultures treated with the non-pathogenic strain of Fusarium when compared to the untreated control. The potential application of the non-pathogenic strain of *F. oxysporum* for enhancing the synthesis of desired secondary metabolites in plant tissue cultures is discussed.

## 1. Introduction

*Linum usitatissimum* L. (flax) is a valuable plant cultivated since ancient times. This plant still draws the interest of researchers and farmers, and hence new flax cultivars are constantly being created. For example, in the last few years, various cultivars of flax have been registered in Poland, including the cultivars Hera (in 2023) and Silesia (in 2020).

Flax is known as a source of fibre and oil. Flax fibres are used by the textile and composite industry, while linseed oil has a wide range of applications in medicine, pharmacology and cosmetics production. This precious plant can be used in biotechnological in vitro methods and cultured in tissue cultures.

Plant tissue cultures are a tool for biotechnology, but they are also applied for commercial micropropagation of plants (micropropagation industry). This type of plant culture can be used for the production of bioactive compounds [1]. Such cultures have many advantages; they may be carried out independently of the climate, weather or seasons, and, moreover, they are completely sterile, which is very important for industrial applications of plant material, for example, in the pharmaceutical and cosmetic industries. It was also demonstrated that in tissue cultures, plant material is standardized, which is not typical for plant material obtained from the natural environment or field cultivation [2]. Plant tissue cultures may also be used for enhancing the production of bioactive compounds present in plants in low concentrations [3]. There are a few methods used to increase the level of plants’ valuable metabolites: modifications of the plant genome (metabolic engineering, genetic modifications, synthetic biology), techniques involving exposing plant tissue cultures to biotic and abiotic stresses [3], and different light spectra [4]. All of these factors may have a beneficial effect on increasing the biosynthesis of secondary metabolites in plants. The reason for this phenomenon is that plant secondary metabolites play a crucial role in plant defence and these compounds are synthesized in response to stresses: biotic stresses, e.g., pathogen infections, as well as abiotic stresses [5]. 

It was previously observed that the non-pathogenic strain of *Fusarium oxysporum* Fo47 had beneficial effects on plant resistance and biocontrol properties on plant pathogens [6]. It was noted that the early response of plant cells to the non-pathogenic strain of *Fusarium oxysporum* Fo47 was more effective than in the case of the pathogenic *Fusarium* strain [7]. Thus, the synthesis of H_2_O_2_ and Ca^2+^ induced by the Fo47 strain was much more intense than that observed for plants inoculated with the pathogenic Foln3 strain. It was suggested that endophytic microorganisms may play a role in the adaptation of plants to biotic and abiotic stresses [8]. The endophytic fungi are classified into four classes of endophytes depending on their colonization and transmission [8]. Fungi from the genus *Fusarium* belong to the second class of endophytes, which exhibit a broad host range, with colonization of stems, roots and rhizomes, and low biodiversity; they may result in an increase in plant biomass, as well as improved resistance of plants (e.g., to salt stress) [9]. The use of a non-pathogenic strain of *F. oxysporum* in the culture of plants (e.g., cucumber) induced an increased response of plants to pathogen (*Pythium*) attack [10]. The protective role of Fo47 was also reported for tomatoes infected with *F. oxysporum* f. sp. *lycopersici* [6]. The suggested mechanism for the biocontrol activity of Fo47 is different from that characterized as “cross protection”. Non-pathogenic strains of *F. oxysporum* Fo47 more efficiently compete for nutrients than pathogenic strains and induce the first stages of plant resistance [11]. The mechanism of endophyte *F. oxysporum* strains relies on the induction of a plant immune response, called an endophyte-mediated response (EMR) [6,12].

Thus, different effects on plant physiology and metabolism have been described for non-pathogenic and pathogenic fungi. The main difference is that Fo47 colonizes mainly the outer part of roots while the pathogenic strains colonize vessels [13]. The result of this fact is a much lower fungal biomass observed in the colonization of non-pathogenic strains when compared to pathogenic strains [14,15]. The second result is that *F. oxysporum* Fo47 does not protect the plants against fungi, which attack the leaves [12].

The subject of this research is flax. In the case of this plant’s elicitation strategy, a yeast extract in a cell culture was used and resulted in the improved synthesis of biologically active compounds (lignans, neolignans) [16]. However, no report is available on the application of non-pathogenic fungi as elicitors in flax suspension cultures. Thus, the aim of the present study was to evaluate the effect of the non-pathogenic *F. oxysporum* strain Fo47 on the biochemical and structural properties of established callus liquid cultures. The explants for callus initiation were derived from two flax cultivars (the oily cultivar Bukoz and the fibre cultivar Nike).

The proposed application of this non-pathogenic fungal strain in flax suspension cultures might serve as a new method for metabolism modification and the induction of desirable bioactive compounds. On the other hand, the applied strategy provided interesting data about the response of plants to a non-pathogenic fungal strain. 

The aim of the present study was to evaluate the impact of a non-pathogenic *Fusarium* strain (*F. oxysporum* Fo47) on the metabolites and structural properties of main cell wall polymers in flax suspension cultures.

## 2. Results and Discussion

### 2.1. Determination of Biocontrol Properties of F. Oxysporum Fo47

**Figure 1:**
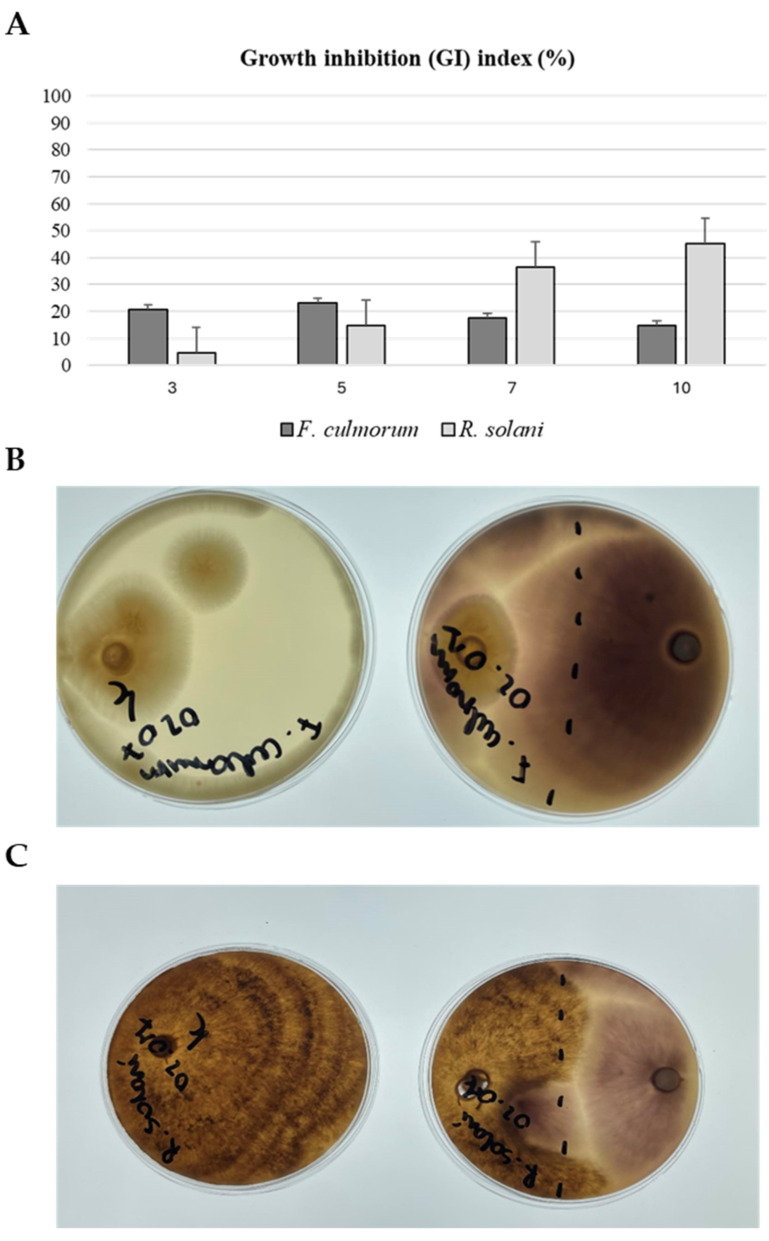
Determination of biocontrol properties of *F. oxysporum* Fo47. (**A**) Growth inhibition (GI) index assessed for a common culture of non-pathogenic strain Fo47 and two pathogenic fungi: *F. culmorum* and *R. solani*. The measurements were performed at 3, 5, 7 and 10 days after inoculation. (**B**) Common culture of the fungi Fo47 and *F. culmorum* after 10 days of inoculation (10 DAI); the Petri dish presented on the left is a control culture of *F. culmorum*. (**C**) Common culture of the fungi Fo47 and *R. solani* after 10 days of inoculation (10 DAI); the Petri dish presented on the left is a control culture of *R. solani*.

In order to analyze the biocontrol properties of the tested *F. oxysporum* Fo47 strain against plant pathogenic fungi (*Rhizoctonia solani*, *Fusarium culmorum*), common cultures were performed as described in the Section 3 (Figure 1). The highest value of the growth inhibition index was calculated for *R. solani* 10 days after inoculation. The biocontrol effect of *F. oxysporum* Fo47 was also observed for *F. culmorum*, but the inhibition index was lower (Figure 1).

### 2.2. Determination of Biomass in Callus Suspension Cultures

**Figure 2:**
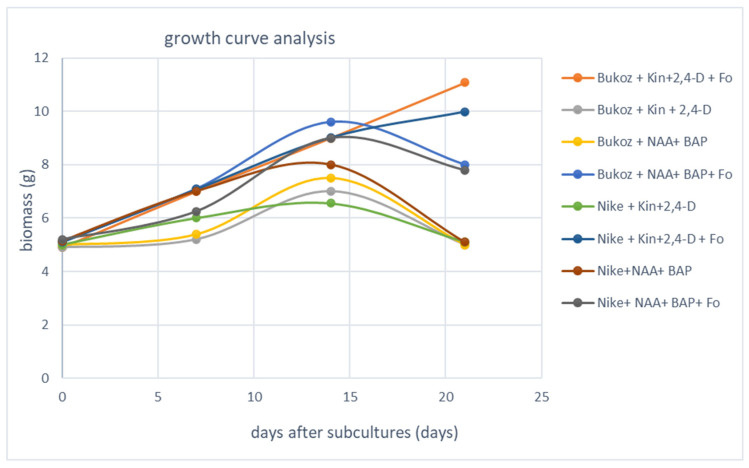
Determination of biomass in flax suspension cultures of two tested flax cultivars (Bukoz, Nike) in various media compositions. Additionally, the cultures were elicited with a non-pathogenic strain of *F. oxysporum* Fo47 and compared to control cultures; this was performed in the same conditions and media but without fungi.

Flax suspension cultures derived from two cultivars of flax, oily Bukoz and fibre Nike, were carried out for 21 days. The cultivar Nike was selected due to its use in the genetic engineering of flax and because many transgenic lines have been generated based on this cultivar [17,18]. Bukoz was chosen because it belongs to the oily cultivar, characterized by high regenerative and morphogenetic abilities in tissue cultures. On the seventh day of the culture, an elicitor (the non-pathogenic strain of *F. oxysporum* Fo47) was added to two types of cultures derived from the two flax cultivars (Nike and Bukoz). It was observed that elicitation caused an increase in biomass growth (Figure 2). All the cultures showed an increase in biomass until the fourteenth day of culture, when the cultures reached the stationary phase of growth. Then biomass was reduced, with the exception of Nike and Bukoz callus cultures grown in media with kinetin and 2,4-dichlorophenoxyacetic acid (2,4-D) and elicited with the non-pathogenic strain of *F. oxysporum*. For these two cultures, a further increase of biomass was noted. These results are in agreement with the data observed for cotton, eggplant and watermelon after treatment with the strain Fo47 by Zhang et al., 2018 [19]. In that paper, a statistically significant increase in root length, leaf area and dry weight (DW) of cotton and watermelon inoculated with an endophytic Fo strain was described. An increase in plant biomass was also observed for *Arabidopsis thaliana* plants inoculated with Fo47 [20]. The suggested reason for this phenomenon might be the induction of nitrogen metabolism by the non-pathogenic strain Fo47 [20].

### 2.3. Content of Photosynthetically Active Pigments in Flax Suspension Cultures

**Figure 3:**
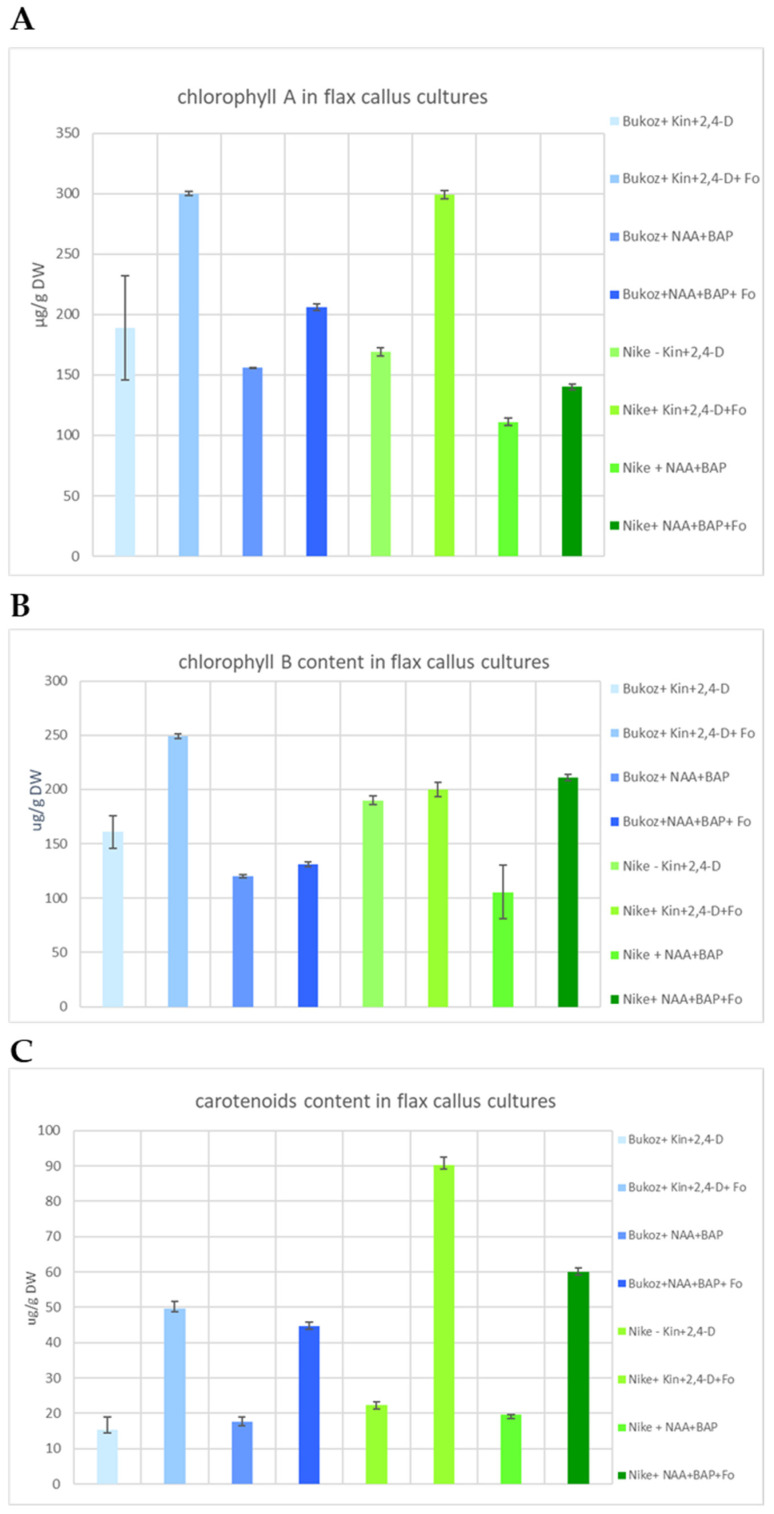
The effect of different media compositions and flax cultivars on the content of photosynthetically active pigments: chlorophyll a, chlorophyll b and carotenoids. Values represent the mean ± SE of three samples.

Flax tissue derived from both analyzed flax cultivars (i.e., Bukoz and Nike) in a medium with the addition of kinetin and 2,4-dichlorophenoxyacetic acid (2,4-D) and the elicitor (non-pathogenic strain of *Fusarium oxysporum* Fo47) showed the highest value of chlorophyll a (300 µg/g dry weight [DW]) (Figure 3A). The suspension culture of the cultivar Bukoz, performed in the above-described conditions, also exhibited the highest level of chlorophyll b (250 µg/g DW) (Figure 3B). It can be assumed that the addition of the elicitor (the non-pathogenic strain of *F. oxysporum*) caused the elevated content of chlorophyll a and b; this relationship was observed in all performed cultures, although their conditions (composition of media, plant material) were different.

It can also be observed that the composition of the medium is crucial for an optimized suspension culture. The application of medium with kinetin and 2,4-D instead of NAA and BAP caused a higher accumulation of photosynthetic pigments (chlorophyll a, b) in plant tissue. These metabolites indicate that analyzed plant tissues were in better physiological conditions than untreated; thus, the promoting influence of the non-pathogenic fungus Fo47 on plant metabolism was confirmed.

In the case of carotenoid content, a similar observation was noted (Figure 3C). Thus, the elicitation of flax suspension cultures with *F. oxysporum* Fo47 increased the accumulation of carotenoids by about 2.5-fold to 3-fold in all cultures except cultures of Nike in media with kinetin, 2,4-D and *F. oxysporum* Fo47. These cultures showed about a 4-fold increase in the amount of carotenoids. Thus, the addition of non-pathogenic fungi stimulated carotenoid synthesis in flax tissue. Carotenoids play an important role in photosynthesis and photoprotection in plants [21], but they are also signalling molecules in pathogenesis and are synthesized in response to changing environmental conditions [22]. It was reported that the stage of plant development also affects the carotenogenesis process [22]. Thus, observed changes in the amount of carotenoids after elicitation with the fungus *F. oxysporum* may result from the plant response to the fungus.

### 2.4. Content of Polyphenols and Flavonoids in Flax Suspension Cultures

Suspension cultures are often treated as a source of natural bioactive compounds, and elicitation is one of the methods used to increase the level of these compounds. For this reason, in established suspension cultures treated with *F. oxysporum* Fo47 elicitation, the content of polyphenols and flavonoids was determined and compared to non-treated cultures.

**Figure 4:**
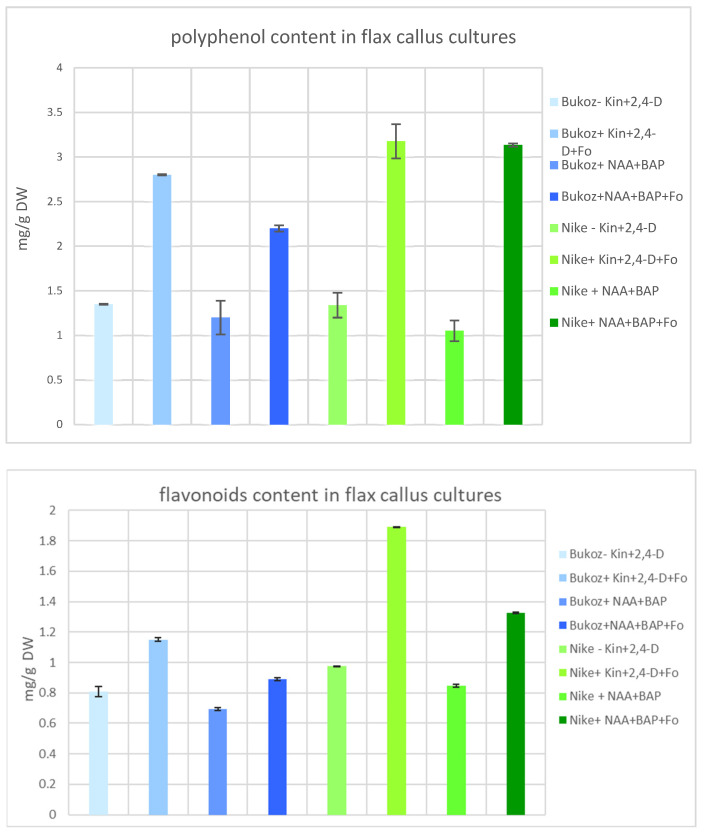
The effect of different media compositions and flax cultivars on the accumulation of bioactive compounds: polyphenols and flavonoids. Values represent the mean ± SE of three samples.

The highest level of polyphenols and flavonoids was noted for the culture derived from the Nike cultivar with kinetin and 2,4-D and elicited with *Fusarium* (Figure 4). In this case, about a 2-fold increase was observed for both polyphenol and flavonoid levels in the culture after the addition of the *Fusarium* elicitor when compared to the culture performed in the same conditions without any elicitor. To summarize, in each tested culture, the addition of *F. oxysporum* Fo47 caused an increase in the content of studied metabolites.

The observed data are in agreement with those observed for pepper plants inoculated with *F. oxysporum* Fo47, for which an elevated amount of caffeic acid in roots was measured [23].

It should be pointed out that phenylpropanoids are involved in the fortification of plant cell walls in avocado tissue cultures, and their role in inducing somatic embryogenesis in those cultures was suggested [24].

The observed induced biosynthesis of phenylpropanoids in flax suspension cultures after inoculation with the non-pathogenic strain Fo47 confirmed the observation that the increased level of these metabolites is correlated with different plant biotic and abiotic stresses and may be induced by different external agents [25].

It was also reported that the level of flavonoids was correlated with the improved resistance of flax to *Fusarium* attack [26].

### 2.5. Spectroscopic Analysis of Flax Suspension Cultures Elicited with F. Oxysporum Fo47

**Figure 5:**
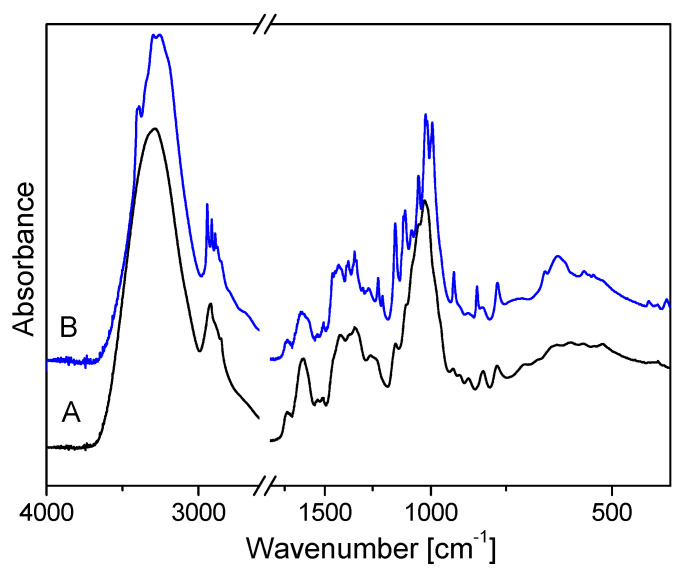
IR spectra of selected samples of calluses measured in the range 400–4000 cm^−1^. The quantitative conclusions from these data were obtained using an internal standard: the band’s intensity at 2850 cm^−1^, which corresponds to νs(CH3) vibrations. The samples were derived from two selected liquid cultures of the Nike cultivar: A—the control culture Nike-C (Nike+kin+2,4-D) and B—Nike-Fox (Nike+kin+2,4-D+*F. oxysporum* Fo47) samples.

The flax tissue of one tested cultivar (Nike) derived from the cultures grown in optimized conditions (Nike+kin+2,4-D) after elicitation with the non-pathogenic strain of *F. oxysporum* Fo47 and the control tissue of the Nike cultivar (without the addition of fungi) were selected for FTIR analyses (Figure 5).

**Figure 6:**
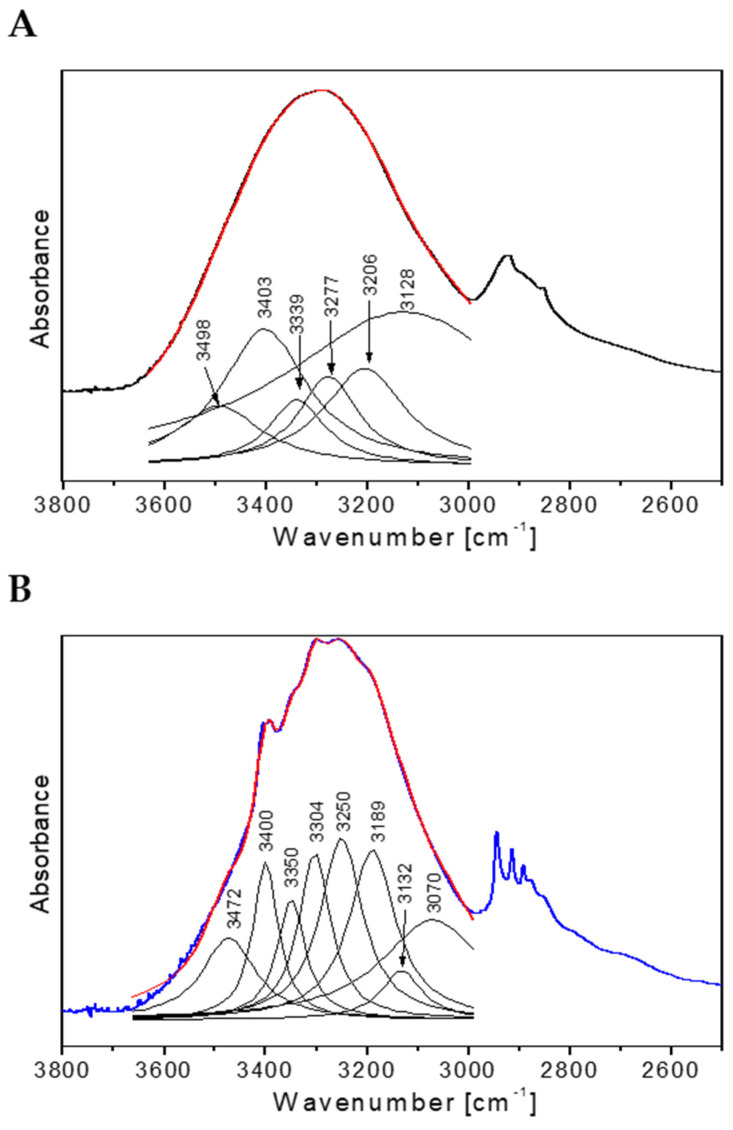
Deconvolution of the IR contours of the tested calluses: (**A**) the control callus Nike-C and (**B**) the tested sample Nike-Fox after treatment with *F. oxysporum* Fo47.

In the FTIR spectra, the broad absorption bands observed in the range 3700–3000 cm^−1^ are defined as bands connected with hydroxyl groups involved in forming hydrogen bonds (intramolecular and intermolecular) (Figure 6). For the control culture (Nike-C), the broad band consists of six Lorentz components and, for the tested callus after treatment with *F. oxysporum* Fo47 (Nike-Fox), it consists of eight components (Figure 6A,B). The bands at about 3500 cm^−1^ correspond to free hydroxyl groups stretching the ν (OH) mode. The Nike-C spectrum contains a component in this range, suggesting the presence of free hydroxyl groups in this sample. The 3480–3340 cm^−1^ range components correspond to the intramolecular hydrogen bonds in cellulose. In this region, two Lorentz components are observed for Nike-C (3403, 3339 cm^−1^) and three for Nike-Fox samples (3472, 3400, 3350 cm^−1^). These data prove that Nike-Fox contains additional intramolecular hydrogen bonds. For Nike-Fox, the component occurring at wavenumber 3400 cm^−1^ is clearly outlined within the broad contour. The following bands, which appear at about 3310–3200 cm^−1^, correspond to intermolecular hydrogen bonds in cellulose [27,28,29,30]. In this region, two Lorentz components are observed for both samples. The integral intensities of the respective components are similar. This suggests a similar content of intermolecular hydrogen bonds in the studied samples. Analyzing the 3200–3000 cm^−1^ range of the FTIR spectra, one additional component for the Nike-C spectrum (3128 cm^−1^) and three components for the Nike-Fox spectrum (3189, 3132, 3070 cm^−1^) can be noted, suggesting the formation of new hydrogen bonds (Figure 6). The FTIR spectrum in the range 3700–3000 cm^−1^ shows that polymers are more strongly bound in the Nike-Fox sample than in the control sample.

Valuable conclusions can be drawn from the analysis of the bands observed at about 1200 cm^−1^ and at about 600 cm^−1^. The first band corresponds to stretching vibrations in the plane of the cellulose monomer ring, coupled with bending vibrations in the O-H···O plane. The second band is characteristic of bending out-of-plane hydrogen bond vibrations. The intensities of these bands are higher for Nike-Fox than for the control sample. The bands at about 1150 and 990 cm^−1^ originate from the ν(COC) vibration of cellulose chains [31,32,33]. The intensities of these bands are higher in the Nike-Fox sample. These data confirm the results of the FTIR spectrum analysis in the range 3700–3000 cm^−1^, characteristic of hydrogen bond vibrations. They prove that the cellulose chains of the Nike-Fox sample are less flexible than those in the control sample.

**Figure 7:**
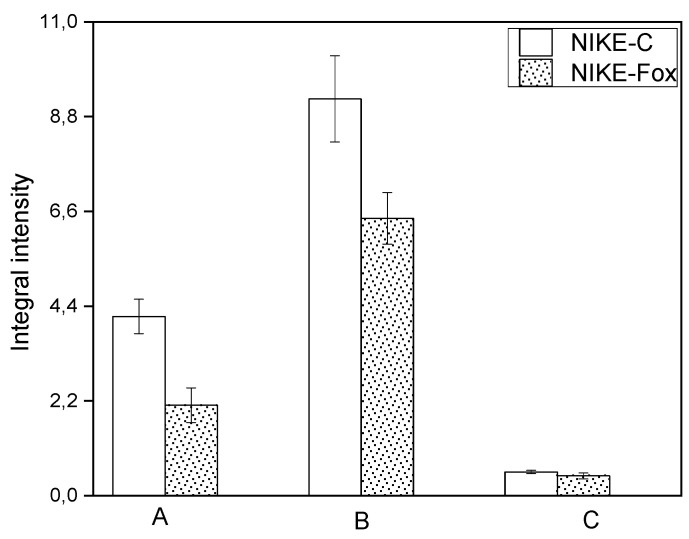
Differences in the integral intensities of the bands at 1457 (A), 1372 (B) and 898 cm^−1^ (C) analyzed in the control callus sample Nike-C and tested sample Nike-Fox after treatment with non-pathogenic *F. oxysporum* Fo47.

The general shape of the FTIR spectrum of Nike is typical of the cellulose spectrum, which is enriched with lignin and pectin bands. Individual bands can be attributed to characteristic cellulose vibrations (φ−glucose ring): δ(CH) + ω(CH_2_) + δ(OH)–1457 cm^−1^; δ_as_(CH_3_, CH_2_)–1429 cm^−1^, δ_s_(CH_3_, CH_2_)–1370 cm^−1^; δ(CH) + δ(OH)–1319 cm^−1^; and ν(C-C) and ν(C-O) in the range of 1200–1300 cm^−1^, δ(φ-OH) at 1163 cm^−1^, ν_as_(C-O-C) in the range of 1000–1110 cm^−1^, γ(CH) in the range 850–1000 cm^−1^ and δ(φ) in the range of 500–720 cm^−1^ [31,32,33]. The integral intensities of bands characteristic for cellulose vibrations are higher for the control than for the Nike-Fox sample (Figure 7).

**Figure 8:**
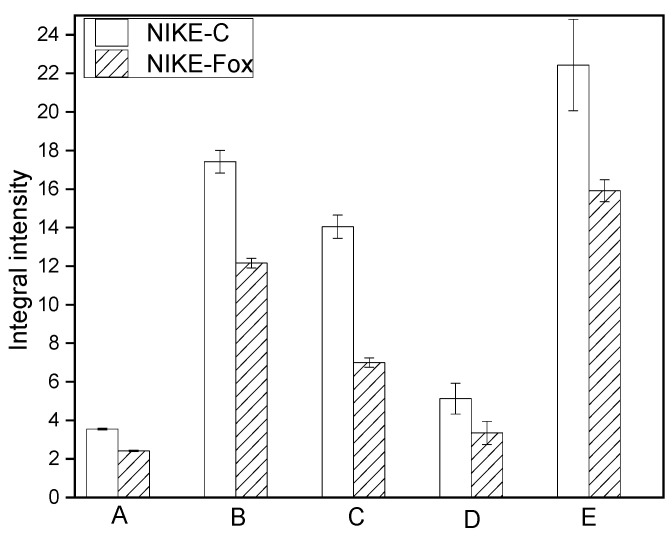
Differences in the integral intensities of the bands at 1735 (A), 1647 (B), 1609 (C), 1104 (D) and 1020 cm^−1^ (E) for the samples of control culture Nike-C and the tested callus after elicitation with Fo47 (Nike-Fox).

Analysis of the IR spectra of plant samples (fibres) in the 1600–1800 cm^−1^ range is used to characterize pectin in plant material [17]. The three components occur at the wavenumbers 1735, 1647 and 1609 cm^−1^ and correspond to vibrations of the carboxyl group present in pectin [34]. The absorption bands at 1104 and 1020 cm^−1^ in the finger-print region (1200–800 cm^−1^) are typical of pectin polymers [35]. The integral intensities of these bands observed in the FTIR spectrum of the control are higher than for the Nike-Fox sample (Figure 8). These data suggest that Nike-Fox contains a smaller amount of pectin in comparison with Nike-C.

**Figure 9:**
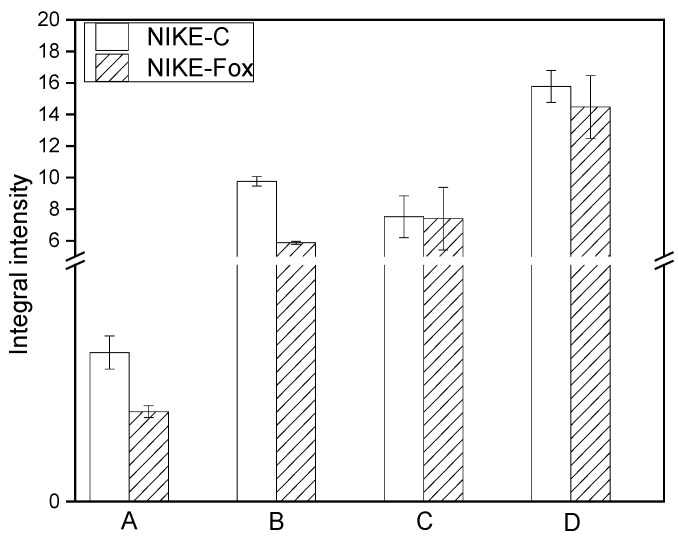
Differences in the integral intensities of the bands at 1545 (A), 1508 (B), 1336 (C) and 1260 cm^−1^ (D) measured for the control sample (Nike-C) and the tested callus after treatment with the non-pathogenic strain of *F. oxysporum* Fo47 (Nike-Fox).

The bands characteristic of vibrations of aromatic lignin rings appear at about 1545, 1508, 1336 and 1260 cm^−1^ [29]. Analyzing the integral intensities of these bands for the studied samples, it can be concluded that Nike-Fox contains less lignin than Nike-C (Figure 9).

The crystallinity index of cellulose (Icr) was calculated as the integral intensity ratio of the 1372 and 2920 cm^−1^ IR bands [36]. The following values compare the Icr parameters for the studied samples: Nike-C: 0.97 and Nike-Fox: 1.13. This means that the Icr values were 15% higher for the Nike-Fox samples compared to the control samples. It can be concluded that the use of non-pathogenic fungi caused the plant’s defence response, manifested by a higher cellulose crystallinity index.

It should be pointed out that the whole flax plant, after inoculation with Fo47, exhibited slightly lower levels of cellulose [14]; a similar effect was observed in the present study in flax suspension cultures. Regarding pectin and lignin levels, no statistically significant changes (except for one line after 48 h of fungal treatment) were observed in whole flax plants in the article by Wojtasik et al., 2024 [14]. The crystallinity index was not determined in whole seedlings, so it is not possible to compare this to data obtained in the present research.

## 3. Materials and Methods

### 3.1. Plant Material

The plant material used in the study was flax *Linum usitatissimum* L. of two cultivars: fibrous Nike and oily Bukoz. The flax seeds were collected from the Institute of Natural Fibres in Poland. The seeds were sterilized in 50% PPM (plant preservative mixture) for 10 min and germinated on MS medium (Murashige and Skoog, 1962) with 1% sucrose and solidified with 0.8% agar. Before sterilization, the pH of the medium was adjusted to 5.8, then the medium was autoclaved at 121 °C for 20 min. The medium used for germination did not contain plant growth regulators. All the procedures were performed in a laminar flow cabinet and plant preservative mixture (375 μL/0.5 L) was added to the medium to prevent microbial contamination. The germination and plant cultures were held in the climatic chamber in constant conditions of growth: relative humidity 60% at a temperature of 21 °C/16 °C in the regime 16 h day/8 h night. The germination step was conducted for 14 days in the dark. Young and healthy seedlings were used for callus initiation.

### 3.2. Induction of Callus

Fragments of hypocotyls and cotyledons of young, sterile seedlings were used for callus initiation. Two different media were used for callus induction: CIM I contained MS medium, 2.5% glucose, 2.5% sucrose, 0.05 mg/L naphthalene acetic acid (NAA), 1 mL/L 6-benzylaminopurine (BAP) and was solidified with 0.8% agar [37]. The pH of the CIM I medium was adjusted to 5.8, and the medium was autoclaved at 121 °C for 20 min. The alternatively used medium, CIM II, contained: MS, 2.5% glucose, 2.5% sucrose, 0.5 mg/L 2,4-dichlorophenoxyacetic acid (2,4-D), 0.5 mg/L kinetin (Kin) and 0.8% agar. Prior to sterilization, the pH of the medium was adjusted to 5.8. Autoclaving was performed as described above.

### 3.3. Fungal Material

The non-pathogenic strain of *Fusarium oxysporum* (ATCC number: MYA-1198, Fo47) was obtained courtesy of the Department of Genetic Biochemistry, University of Wroclaw, and *R. solani* was obtained from the Department of Plant Protection, Wrocław University of Environmental and Life Sciences The fungal strains were cultured on potato dextrose agar (PDA) at 26 °C in the dark.

### 3.4. Growth Inhibition Tests of Plant Pathogens

The antagonistic ability of *F. oxysporum* Fo47 against *F. culmorum* and *R. solani* was assessed using the dual-culture test. Mycelial discs (10 mm) of the pathogens were placed on the edge of PDA plates and, opposite them at a distance of 1.5 cm from the edge, a mycelial disc of the non-pathogenic strain of *F. oxysporum* Fo47 was placed. The plates were incubated at 25 ± 2 °C for 10 days. The growth of fungi was measured daily, and the percentage of growth inhibition (GI) was calculated relative to the control. All the calculations were performed according to the equation [38]:

$$GI=\frac{D1-D2}{D1}\times 100\%$$

D1–diameter of the pathogen colony in control; D2–diameter of the pathogen colony in treatment.

### 3.5. Callus Suspension Cultures

Initiated calluses were cultured in liquid media of two types. CSC I (callus suspension culture I) contained: MS medium, 3% sucrose, 1 mg/L BAP, 0.05 mg/L NAA and 375 μL/0.5 L PPM. Plant growth regulators were added after medium sterilization in an autoclave at a temperature of 121 °C for 20 min.

The second tested medium, CSC II (callus suspension culture II), contained MS, 3% sucrose, 0.5 mg/L kinetin, 0.5 mg/L 2,4-D and 375 μL/0.5 L PPM solution.

### 3.6. Plant Treatment with F. Oxysporum Strain Fo47

Seven-day-old suspension flax cultures (performed in the medium without PPM) were inoculated with a 1 mL microconidial suspension of the tested strain *F. oxysporum* Fo47 (10^9^ conidia/mL); in the control cultures, no elicitation was used.

### 3.7. Analysis of FTIR Spectra

All spectra were processed in the same way before statistical analysis. In the first step, the spectra were compared and analyzed by commercial computer software (OriginPro 2024, OriginLab Corp., Northampton, MA, USA). This analysis included background subtraction and deconvolution of the experimental bands into the Lorentz components.

## 4. Conclusions

The obtained data indicate that the non-pathogenic strain of *F. oxysporum* Fo47 can be successfully used to elicit liquid callus cultures. This method allows one to increase the content of secondary metabolites in plant tissues. In this study, two different media compositions were also optimized, and it was found that the liquid MS medium with the addition of kinetin and 2,4-D after supplementation of the culture with the non-pathogenic strain of *F. oxysporum* Fo47 resulted in the highest callus biomass. These cultures also remained in the logarithmic growth phase longer than other tested liquid cultures. The optimized cultures exhibited an increase in the amount of photosynthetically active pigments and about a 2-fold higher accumulation of bioactive compounds (polyphenols, flavonoids) than control cultures (without fungi). The application of the non-pathogenic *F. oxysporum* Fo47 strain also resulted in changes of cellulose structural properties. A higher cellulose crystallinity index (about 15% higher) as well as a higher level of phenylpropanoids was observed for cultures treated with *Fusarium* Fo47 when compared to non-treated control cultures. Thus, it is suggested that plant cell walls were fortified with higher crystallinity cellulose and increased levels of phenylpropanoids after inoculation with endophytic Fo47.

Finally, it can also be concluded that the cultivar Nike seems to be more efficient, especially in terms of the synthesis of bioactive compounds, when compared to the other tested flax cultivar Bukoz. In the near future, further analyses will be carried out to determine levels of individual metabolites, especially from the class of phenolic compounds, in flax suspension cultures after treatment with the non-pathogenic *F. oxysporum* strain. It will also be interesting to increase the scale of flax suspension cultures in optimized conditions, which were established in the present study, and to analyze the level of secondary metabolites. Another direction of future research will be the establishment of suspension cultures from genetically modified flax plants, synthesizing PHB (poly-β-hydroxybutyrate) [37], inoculation with a non-pathogenic strain of *F. oxysporum* Fo47 and analyses of metabolites with biomedical potential (PHB, KYNA, phenolic compounds etc.).

## Data Availability

Data is contained within the article.
